# Concurrent Primary Cutaneous Cryptococcosis and Bowen's Disease in a Patient With Multiple Sclerosis on Fingolimod: A Rare Case Report and Review of Literature

**DOI:** 10.7759/cureus.71050

**Published:** 2024-10-08

**Authors:** Gullu Gencebay, Nisan Çetin, Melin Gecer, Ozlem Su Kucuk

**Affiliations:** 1 Department of Dermatology, Bezmialem Vakif University Faculty of Medicine, Istanbul, TUR; 2 Department of Pathology, Bezmialem Vakif University Faculty of Medicine, Istanbul, TUR

**Keywords:** bowen disease, fingolimod, multiple sclerosis, primary cutaneous cryptococcosis, treatment

## Abstract

Fingolimod is an oral disease-modifying treatment used for the relapsing-remitting forms of multiple sclerosis, which may render patients susceptible to opportunistic infections and lead to an increased risk of skin cancer. We report a 56-year-old woman with multiple sclerosis in remission on fingolimod treatment for five years presenting with the following skin lesions. Our patient presented with a non-healing ulcerated erythematous lesion on the left lower abdomen for six months and a crusted erythematous plaque on the forehead that had not healed for one year. Skin biopsy from the abdominal lesion showed fungal spores extending from the papillary dermis to the deep dermis, mostly in histiocytes and periodic acid-Schiff (PAS) staining revealed structures consistent with *Cryptococcus*. A moderate *Cryptococcus neoformans *infection was confirmed by culture. There were no systemic symptoms suggestive of secondary cutaneous cryptococcosis. In addition, a punch biopsy from the forehead showed findings compatible with Bowen's disease. Fingolimod was discontinued and the lesion on the left lower abdomen resolved with oral fluconazole therapy. Complete surgical excision with negative margin was performed for Bowen's disease. We present the first reported case of concurrent primary cutaneous cryptococcosis and Bowen's disease in the same patient with multiple sclerosis on fingolimod in the literature.

## Introduction

Fingolimod, the first oral disease-modifying therapeutic agent in multiple sclerosis, acts as a sphingosine-1-phosphate (S1P) receptor agonist that results in compromised traffic and recirculation of lymphocytes. It inhibits S1P-mediated lymphocyte exit from lymph nodes impairing peripheral lymphocyte recirculation. This distinct mechanism leads to a decrease in overall lymphocyte counts [1]. Fingolimod acts as an immunomodulatory compound in addition to its acute effects on lymphocyte distribution. Some cases of viral and cryptococcal infections and skin cancers have been reported on fingolimod treatment [2]. Real-life experience with fingolimod treatment includes the development of T-cell lymphoma (mostly cutaneous), malignant melanoma, squamous cell carcinoma, Kaposi's sarcoma and Merkel cell carcinoma [3]. Cryptococcal infections are commonly opportunistic infections caused by fungi from the genus Cryptococcus, which cause significant morbidity and mortality, especially in immunocompromised patients. Primary cutaneous cryptococcosis is a rare localized condition affecting only the skin, resulting from direct fungal inoculation without systemic involvement. However, if not diagnosed early, primary cutaneous cryptococcosis may progress to secondary dissemination, causing fatal meningoencephalitis and other complications [4]. Cutaneous manifestations of primary cutaneous cryptococcosis are polymorphic, such as papules, purpura, vesicles/blisters, pustules, nodules, tumors, ulcerations, necrotizing panniculitis/cellulitis, abscesses, acne-like lesions, and molluscum contagiosum-like lesions, because of that the diagnosis may be delayed and may lead to secondary dissemination [5]. We present our patient with multiple sclerosis on fingolimod treatment for five years and whose biopsy and fungal culture from the ulcerated lesion on the left abdomen grew Cryptococcus neoformans and biopsy from the forehead showed findings compatible with Bowen's disease.

## Case presentation

**Figure 1:**
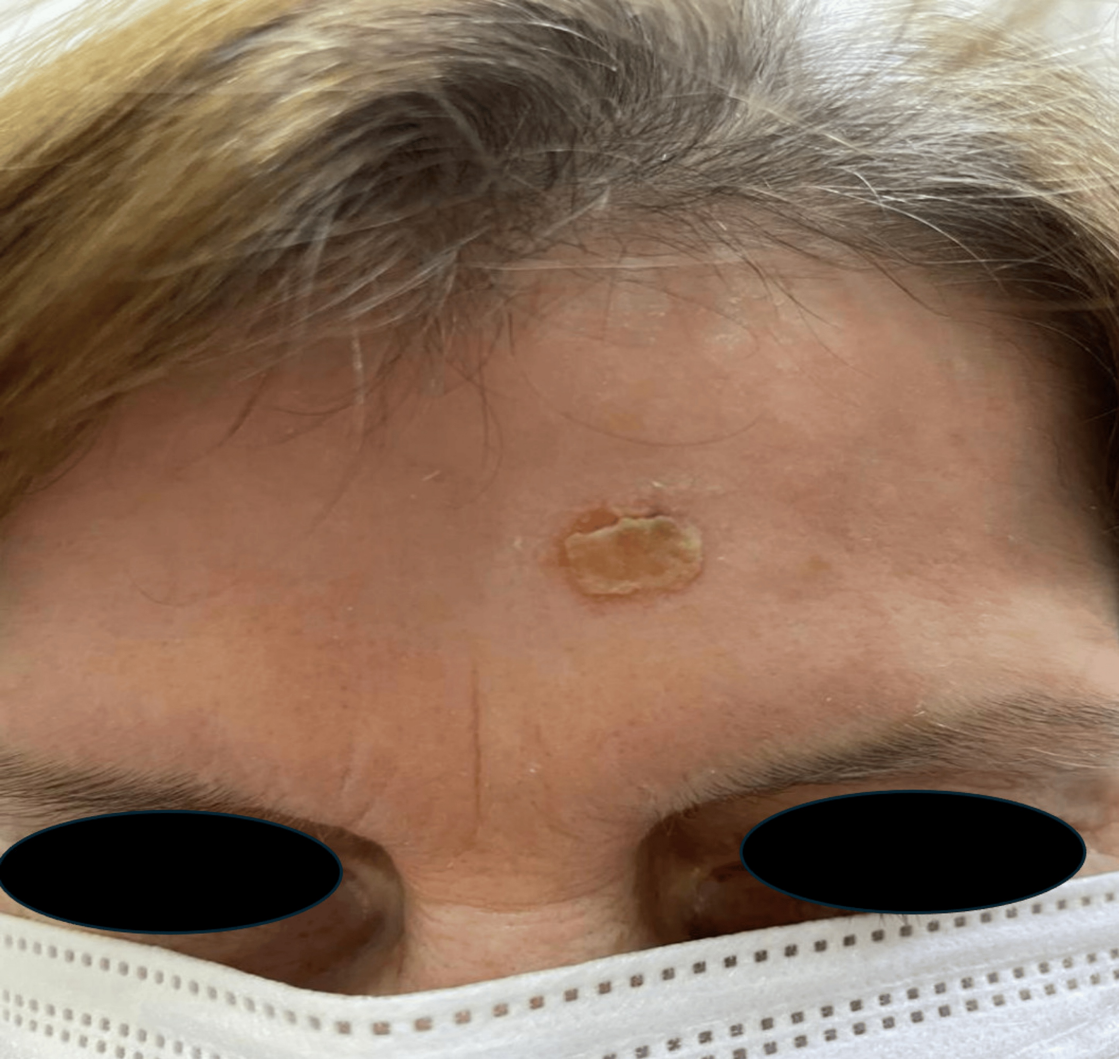
A crusted erythematous plaque on the forehead

**Figure 2:**
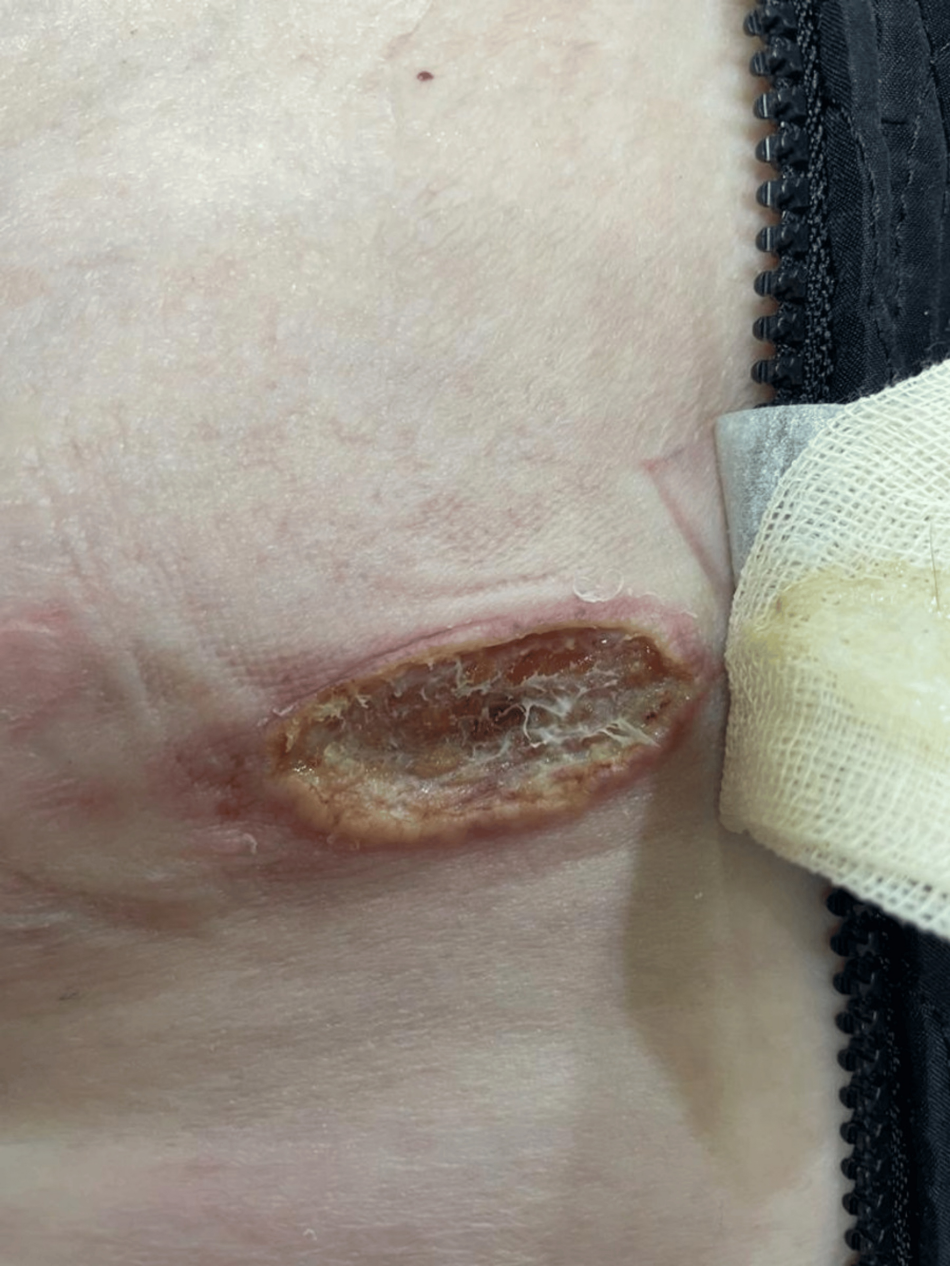
An erythematous ulcer on the left abdomen

A 56-year-old female patient with multiple sclerosis on fingolimod treatment for five years presented to our clinic with the complaint of a wound on the left abdomen for six months and on the forehead for one year. The patient stated that the wound on the forehead occurred after a hot oil splash during cooking, and there was no regression despite the use of topical antibiotic cream for one year; the wound on the abdomen started six months ago as a spontaneous pimple and grew, there was no history of trauma before, there was no regression with topical antibiotic creams, and it expanded gradually. The patient had no fever, night sweats, headache or other neurologic and systemic complaints other than the existing lesions. Dermatologic clinical examination revealed a 2 x 3 cm crusted erythematous plaque on the forehead (Figure 1) and a 5 x 6 cm erythematous ulcer on the left abdomen (Figure 2).

**Figure 3:**
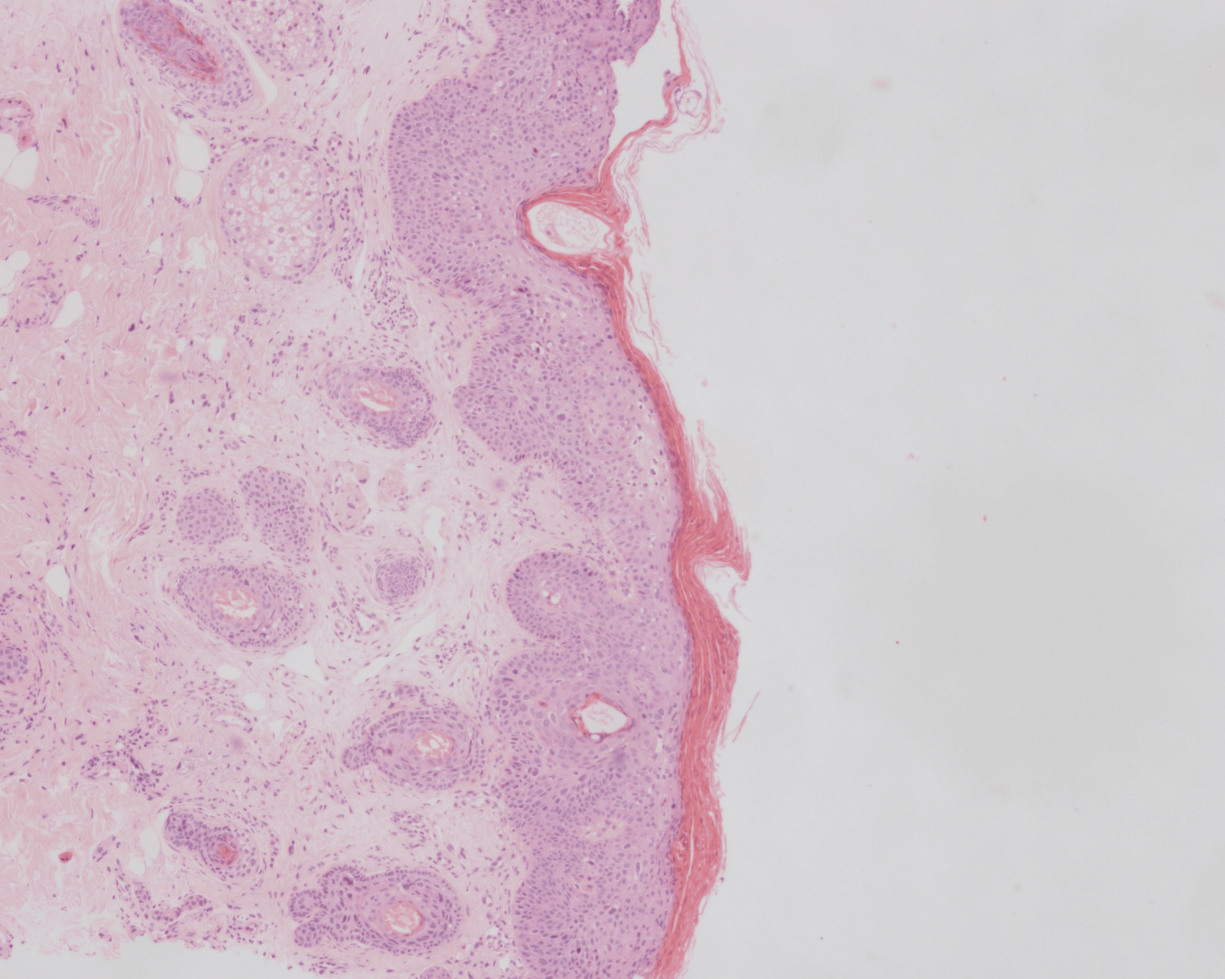
Full-fold dysplasia of the epidermis, H&E x10

**Figure 4:**
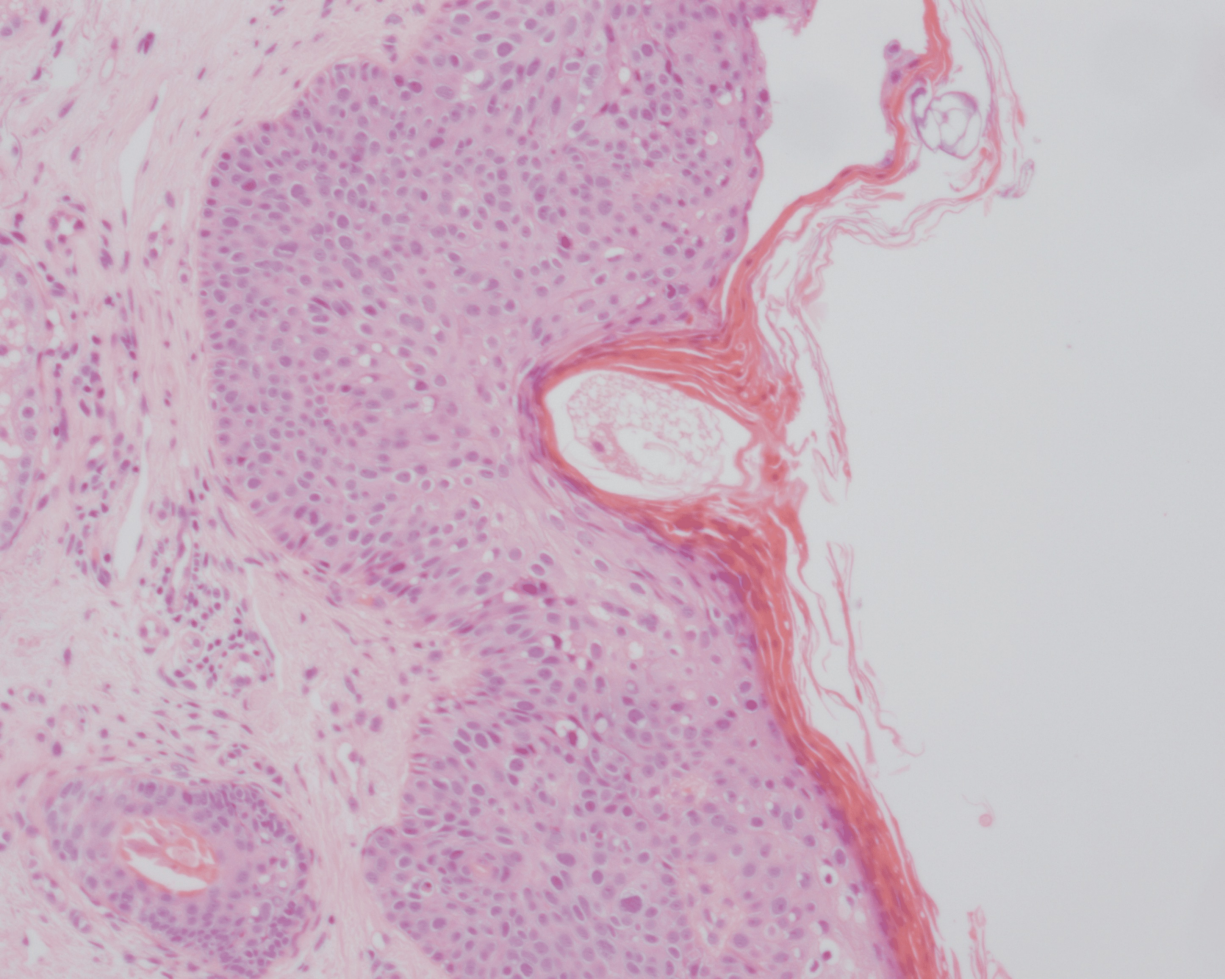
Pleomorphism and mitoses in the epidermis, H&E x10

Skin punch biopsy was performed from both lesions. The lesion on the forehead showed findings compatible with Bowen's disease (Figures 3, 4).

**Figure 5:**
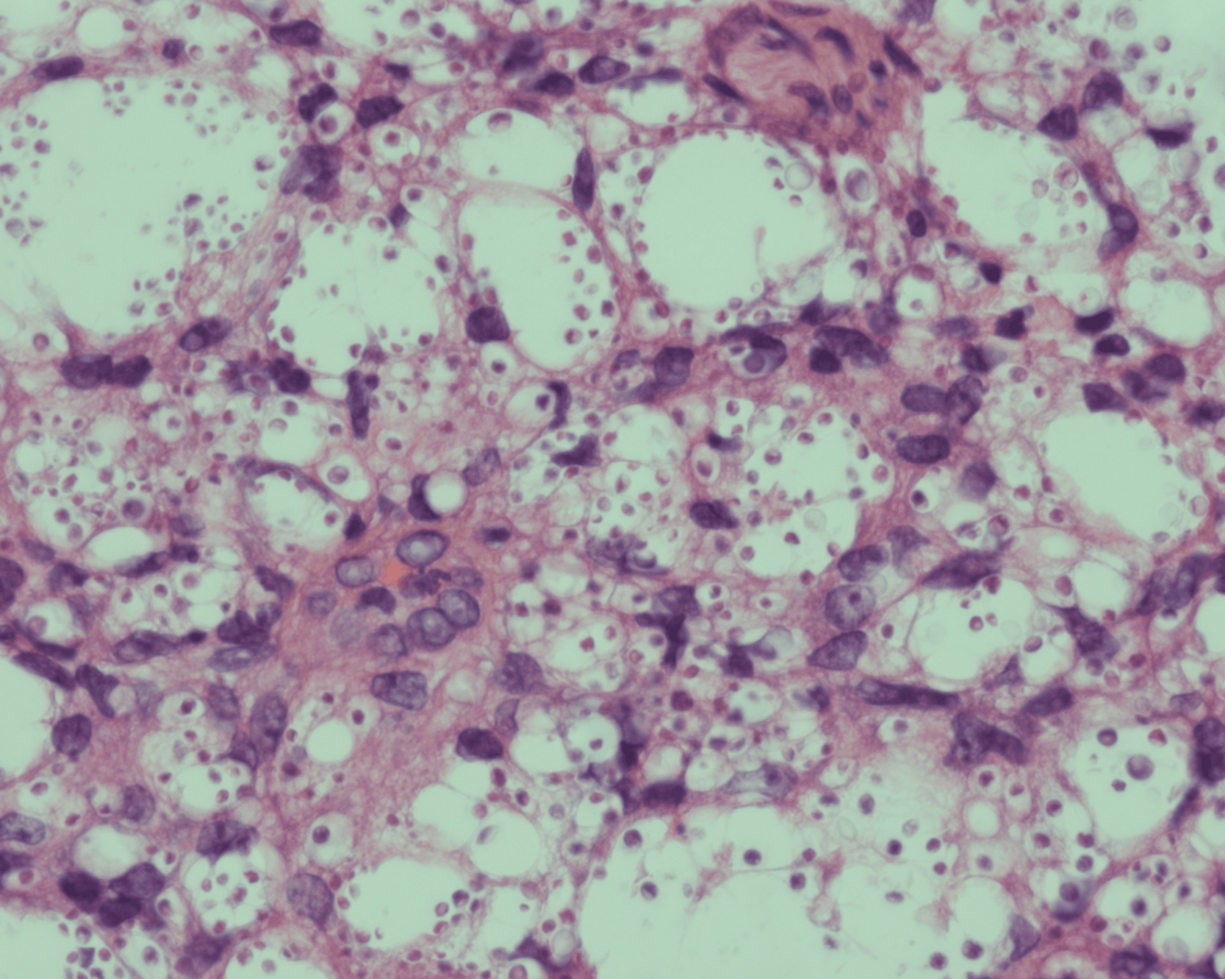
Punch biopsy from the abdomen showing fungal spores starting from the papillary dermis and extending to the deep dermis, mostly in histiocytes, H&E x40

Punch biopsy from the abdomen showed fungal spores starting from the papillary dermis and extending to the deep dermis, mostly in histiocytes (Figure 5).

**Figure 6:**
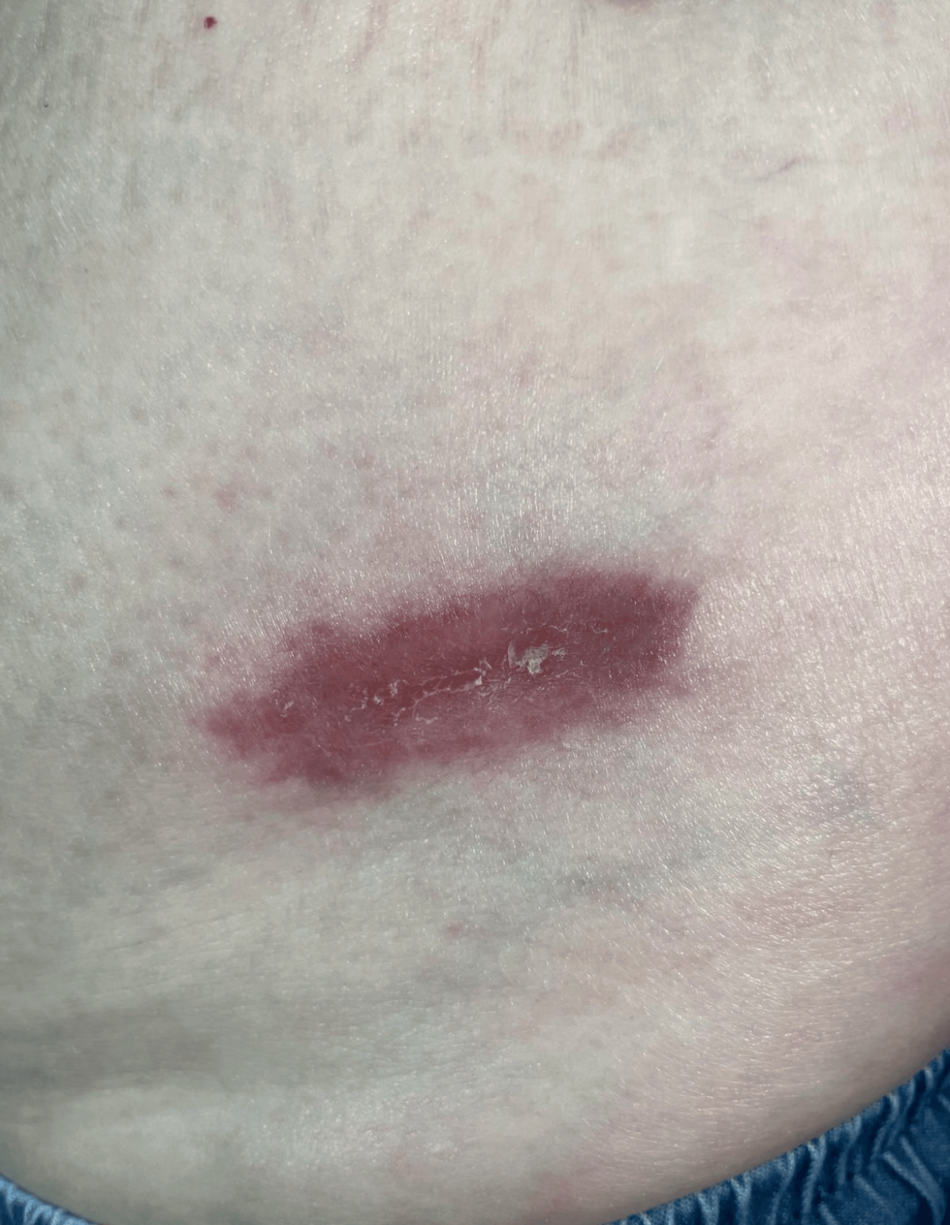
The lesion on the left abdomen completely healed at the end of two months of treatment

Periodic-acid Schiff (PAS) and Wright-Giemsa staining methods were used for identification, and the results were consistent with Cryptococcus. The tissue biopsy culture was obtained from the abdominal ulcer. Culture confirmed a moderate number of C. neoformans. There were no clinical indications for a cerebrospinal fluid analysis and computed tomography scan head given the absence of neurological symptoms. Complete blood counts and a comprehensive metabolic panel were performed and determined within normal limits. Human immunodeficiency virus (HIV), hepatitis C and hepatitis B serology were negative. The patient was consulted by neurology for consideration of an alternative medication for multiple sclerosis. Fingolimod treatment was discontinued and dimethyl fumarate treatment was started for multiple sclerosis. Oral fluconazole 400 mg daily was started. The lesion on the left abdomen was observed to have significantly rapid improvement within the initial two weeks, and it completely healed at the end of two months of treatment (Figure 6).

An excisional biopsy of the lesion on the forehead was planned, and it showed findings compatible with Bowen's disease. Complete surgical excision had a negative margin.

## Discussion

In our patient, concurrent primary cutaneous cryptococcosis and Bowen's disease occurred after five years of fingolimod treatment. Long-term use of fingolimod is known to cause various adverse events. Fingolimod was the first oral disease-modifying therapy approved for relapsing-remitting multiple sclerosis, acting on lymphocyte circulation. Long-term efficacy and adverse events of fingolimod have been investigated in many studies. In a five-year follow-up of a long-term study, adverse events of particular interest were identified, particularly lymphopenia, hypertension, leukopenia, and increased hepatic enzyme levels, which required medical treatment and continuous monitoring. In this study, the authors also specified the occurrence of rare adverse events reported in case reports. Cryptococcal meningitis and progressive multifocal leukoencephalopathy were detected in one patient and two patients, respectively [6].

Fingolimod acts as a disease-modifying therapy for multiple sclerosis by downregulating sphingosine-1-phosphate receptors on lymphocytes. This action leads to the selective retention of C-C chemokine receptor type 7 (CCR7)+ naive T cells and central memory T cells in lymphoid organs. Fingolimod reduces the levels of circulating CD4+ T cells (cluster of differentiation) and, to a lesser degree, CD8+ T cells without affecting antigen presentation, activation, proliferation, differentiation, or effector function of T or B cells. Th1 immune response plays a critical role in controlling fungal infections. Fingolimod modifies the cytokine environment and diminishes subsets of natural killer cells, leading to a shift in the host immune response from T helper 1 (Th1) to Th2 in cryptococcal infections. Over an extended period (51-64 months), the treatment of fingolimod results in a reversal of the typical healthy 2:1 ratio of CD4 to CD8 [7,8]. In a case report of scalp cryptococcosis with lymphopenia (CD4 count was 13 cells/uL) without disseminated cryptococcal infection on fingolimod treatment, it has been recommended that patients should have their CD4 count monitored regularly and serum cryptococcal antigen tested if it is below 100 cells/uL. In our patient, primary cryptococcal infection occurred without lymphopenia. The lymphocyte count was 1.30/μL (range, 1000-5000/μL). However, flow cytometry was not performed, and the CD4/CD8 lymphocyte subset was not evaluated. Although there was no lymphopenia, cryptococcal infection developed as a consequence of the CD4 counts, which may have been low. Therefore, clinical questionnaires related to cryptococcosis and routine dermatological examinations are recommended regularly. The duration of treatment with fingolimod and age are significant risk factors for developing cryptococcal infection [8]. Similarly, the duration of fingolimod treatment was five years in our 56-year-old patient. Our recommendation includes routine monitoring of CD4 counts by flow cytometry in patients on long-term use of fingolimod therapy (at least five years), even without lymphopenia.

C. neoformans infection rarely occurs in immunocompetent patients, while immunocompromised hosts, especially those with advanced HIV/AIDS and severely weakened immune systems, often lead to disseminated disease. The most commonly affected organs are primarily the central nervous system, followed by the lungs and skin, which may present as ulcerations, acne-like lesions, pustules, nodules, abscesses, cellulitis, and molluscum contagiosum-like lesions [9]. Primary cutaneous cryptococcosis is a rare localized cryptococcal infection affecting only the skin without systemic infection. In the PubMed literature search, to our knowledge, it was seen that there were only three other cases diagnosed as primary cutaneous cryptococcosis on fingolimod treatment [7,8,10]. Regarding the cases of primary cutaneous cryptococcosis reported earlier in patients on fingolimod treatment, cryptococcal infection was associated with lymphopenia in all three cases. The CD4 counts were low in two patients [7,8] and were not analyzed in one patient [10]. One case involved a six-month course of fluconazole at 400 mg daily, while the other utilized a six-week regimen consisting of an 800 mg loading dose followed by 400 mg daily. In both cases, fingolimod treatment was discontinued [7,8] while in one case, treatment was continued, and oral fluconazole 600 mg twice a day for 14 days followed by 400 mg twice a day for four months was prescribed [10].

Furthermore, studies reported that fingolimod treatment in multiple sclerosis can be associated with an increased risk of cancer. The literature review was performed using the keywords: "fingolimod", "multiple sclerosis", "fingolimod and skin malignancy", "fingolimod and bowen disease" in the databases of PubMed. Rare reports were published that patients treated with fingolimod developed cutaneous melanoma, basal cell carcinoma, squamous cell carcinoma, Kaposi’s sarcoma, Merkel cell carcinoma, mycosis fungoides, cutaneous large B-cell lymphoma, primary cutaneous CD30+ anaplastic large-cell T-cell lymphoma and lymphomatoid papulosis. In the LONGTERMS study evaluating the safety and efficacy of fingolimod in relapsing-remitting multiple sclerosis patients with up to 14 years of exposure, 12.6% of patients experienced at least one serious adverse event during the study and basal cell carcinoma was one of the most frequently observed serious adverse events [11]. Fingolimod causes the peripheral blood lymphocyte count to decrease to 20%-30% of baseline. Chronic fingolimod intake also leads to a decrease in neutrophil granulocyte count to approximately 80% of baseline. Immunosuppression is known to promote cancer development [12]. It is thought that such lymphocyte depletion may eliminate or neutralize melanoma-specific lymphocytes and inhibit immune surveillance against cancer. Fingolimod, which activates the interleukin-6/janus kinase/signal transducers and activators of transcription-3 pathway (IL-6/JAK/STAT), also has a pro-oncogenic effect [13]. Despite these hypotheses, cases of malign melanoma occurring in patients treated with fingolimod are currently considered to be anecdotal, and there is no significant evidence of an increased risk of malign melanoma. In the LONGTERMS study, an increased risk of malignancy was reported in 1.6% of patients treated with fingolimod. Hematological or solid organ malignancies were rare, whereas skin malignancies, basal cell carcinoma, and squamous cell carcinoma were more common [11]. Brufau-Cochs et al., in a review in 2024, showed that sphingosine-1-phosphate receptor modulators such as fingolimod are associated with a higher risk of basal cell carcinoma but not squamous cell carcinoma or melanoma [14].

## Conclusions

In conclusion, we observed that there is evidence of an increased risk of non-melanoma skin cancer and cryptococcal infection in patients with multiple sclerosis on fingolimod treatment. This study is limited by the small case series reported in the literature; further research is needed to determine the relationship between fingolimod and cryptococcal cases and skin malignancies.

Long-term use of fingolimod has been reported to cause changes in lymphocytes. Therefore, we recommend routine monitoring of CD4 counts by flow cytometry in patients on long-term use of fingolimod therapy (at least five years), even without lymphopenia, and we believe that clinical and dermatoscopic examination should be performed regularly every six months for the development of skin malignancies.
